# 

**Published:** 1888-07

**Authors:** 


					﻿CONTRIBUTORS TO VOL. IV.
Dr. A. M. Autrey . .............................Houston
Prof. Odo Betz, M. D..................Heilbron,	Germany
Dr. R. H. L. Bibb..................... Saltillo, Mexico
Dr. E. J. Beall...............................Ft. Worth
Dr. T. J. Bennett........  .	.'..................Austin
Dr. S. J. Cunningham.............................Dallas'
Dr. Sam Cunningham................................Elgin
Dr. B. F. Church.................................Austin
Dr. A. N. Denton.................................Austin
Prof. R. H. Day, M. D . . ...............N. O. Polyclinic
Dr. J. F. Eaves................................Wellburn
Dr. H. L. Fontain.................................Bryan
Dr. W. P. Fleming.........*..................Georgetown
Prof. Landon Carter Gray, M. D...........N. Y. Polyclinic
Prof. B. E. Hadra, M. D.......................Galveston
Dr.' W. G. Jameson......................1 . . . . Rusk
Prof. Emory Lamphear, M. D...............Kansas City, Mo
Dr. J. W. McLaughlin.............................Austin
Dr. J. E. Mayfield..........................Nacogdoches
Dr. R. Menger...............................San Antonio
Dr. T. A. Pope.................................Cameron
Dr. D. S, Peeples  ............................Navasota’
Dr. Daniel Parker...............................Calvert
Dr. Jno. Preston...............................  Austin
Dr. H. S. Robertson......................Peytonville, Ark
Dr. W. T. Richmond .............................. Manor
Dr. C. M. Ramsdell.............................Lampasas
Prof. W. B. Rogers, M. D..................Memphis, Tenn
Dr. Bat. Smith................................. Wharton
Dr. R. M. Swearingen............................ Austin
Dr. F. A. Schmitt............... .........-. Schulenburg
Dr. Q.	R.	Shuford ................................Tyler
Dr. R.	P.	Talley...............................  Belton
Dr. T.	J.	Tyner................................. Austin
Dr. D.	R.	Wallace...............................Terrell
Prof. W. H. Wathen, M. D. .	•............Louisville, Ky
Prof. H. A. West, M. D. ............ . Galveston
Dr. T. D. Wooten..........y	........... Austin
Dr. L- B. Underhill.......................New Braunfels
Our Advertisers.—The fall and winter announcements of
several first class Medical Colleges will be found in this issue,
and we advise physicians and students to tead them carefully;
there is something of great interest in each, and they are not
merely formal announcements of the opening and closing of the
sessions. We cannot “ puff” any of them—they do not need it;
and to single out any one or two for special mention would ap-
pear unfair, while it is out of the question that we should give
each, as well as all other new advertisements, a separate editorial
mention. This has become customary, we know; but it is as sense-
less, as it is unjust to the publisher. It is giving up large space
to notices which it is questionable if any read, seeing at a glance
what they are. So we call attention to all of our advertisements;
they constitute a very interesting feature of the Journal.
Among the advertisements will be found also something of
more than ordinary interest from manufacturing chemists—the
several proprietry articles of artificial foods and stimulants, for
instance, which are especially adapted to the season. Read
them all; we are abundantly favored in patronage in this depart-
ment, for which we return here our acknowledgments. In cor-
responding with any of our advertisers, please mention the
Journal.
				

